# 

**Published:** 1881-04

**Authors:** 


| | PAGE |
|---|---|
| ORIGINAL LECTURE—The History of the Horse. By Louis H. Laudy, Ph.D. . . . . . . . . . | 79 |
| ORIGINAL COMMUNICATIONS—Trance and Trancoidal States in the Lower Animals. By George M. Beard, A.M., M.D. . . . . . . . . . . | 90 |
| Irido-Choroiditis, commonly called Moon Blindness, in the Horse. By William O. Moore, M.D. | 106 |
| Veterinary Hygiene and the New York City Stables. By Allan S. Heath, M.D. . . . . . . . | 114 |
| Posterior Paralysis in Mules, from Entozoa in the Kidneys. By G. M. Bowler, M.D., V.S. . . . | 118 |
| EDITORIAL—Government and the Investigation of Contagious Diseases among Domestic Animals . | 121 |
| Ethics in Veterinary Practice . . . . . . . . . . | 125 |
| Ensilage and Disease . . . . . . . . . . | 126 |
| Contributions to the JOURNAL OF COMPARATIVE MEDICINE . . . . . . . . | 127 |
| REVIEWS—Manual of Cattle-Feeding. By Henry P. Armsby, Ph.D. . . . . . . . . | 128 |
| De la Leucocythémie chez les Animaux Domestique Par M. Ed. Nocard . . . . . . . | 129 |
| Vade Mecum of Equine Anatomy. By A. Liautard, M.D., D.V.S. . . . . . . . . | 129 |
| CASE DEPARTMENT—Amputation of Tongue . . . . . . . . . . | 130 |
| Lacerated Wound of the Neck . . . . . . . . . . | 130 |
| Abscess of Hip . . . . . . . . . . | 131 |
| Cystic Change in the Mucous Membrane of the Stomach of a Horse . . . . . . . | 131 |
| Hemiplegia following Influenza, in a Horse . . . . . . . . . . | 133 |
| Hog Cholera on Long Island—Treatment by Chlorate of Potash . . . . . . . . | 134 |
| CORRESPONDENCE—Foot-and-Mouth Disease in the United States . . . . . . . . | 136 |
| PROGRESS OF VETERINARY SCIENCE—Nitrite of Amyl—Composition and Amount of Urine of the Horse—Chloral as a Sedative for Horses—Treatment for Piles in Horses—How to Examine the Horse's Eye—Treatment of "Moon Blindness"—A New Poison in Pork—Treatment of Trichinosis by Glycerine—Treatment of Hog Cholera—A New Disease Developed from Hydrophobic Saliva—Cure for Dog Distemper—Foot Rot—Dyspepsia in Hens—Transmission of Tuberculosis—Diseases of Sheep—Is Charbon Transmitted by Plants and the Soil?—Roup in Fowls—Black-Leg (External Charbon), not Anthrax; Another Protective Virus. . . . . . . . . | 138 |
| NEWS AND MISCELLANY—Mustang Liniment—Bi-Carbonate of Potassium in Rheumatism—A British Congress of Veterinary Surgeons—Social Recognition of Veterinary Surgeons—Veterinary College for Ireland—The New York Aquarium—A New Zoölogical Garden in New York City—Prize for an Essay on Paraplegia in the Horse—Veterinary Medicine in England—Salicylic Acid in Foot-and-Mouth Disease—Death of a Veterinary Surgeon from Strychnine—Foot-and-Mouth Disease in New York City—Analysis of the Haas Hog Cholera Remedy—Strange Cattle Disease—Epidemic of Spinal Meningitis Among Horses—Selling Diseased Meat—Connecticut Veterinary Law—The Hog Cholera and Trichinosis Scare—Rural Physiology—Extent of Pleuro-Pneumonia in the United States—Foot-and-Mouth Disease in England—Does Foot-and-Mouth Disease Exist in this Country?—The Brown Institution—Clarke on the Uses of the Canine Teeth—Congress and Veterinary Medicine—Trichinosis and American Pork—Columbia Veterinary College: Fourth Annual Commencement . . . . . . . . . . | 144—150 |

